# Early results of systematic drug susceptibility testing in pulmonary tuberculosis retreatment cases in Cameroon

**DOI:** 10.1186/1756-0500-5-160

**Published:** 2012-03-21

**Authors:** Jürgen Noeske, Natascha Voelz, Elisabeth Fon, Jean-Louis Abena Foe

**Affiliations:** 1 German Development Cooperation (GIZ), P.O. Box 7814, Yaounde, Cameroon; 2 Regional Tuberculosis Control Unit, Regional Delegation of Public Health, P.O. Box 106, Douala, Cameroon; 3 National Tuberculosis Control Program, P.O. Box 15656, Yaounde, Cameroon

**Keywords:** Drug resistant tuberculosis, retreatment, surveillance, Cameroon

## Abstract

**Background:**

The number of pulmonary tuberculosis (PTB) patients reported with resistance to first-line anti-tuberculosis drugs after a standardized retreatment regimen in Cameroon is increasing. Hence, the National Tuberculosis Control Program (NTP) implemented, in one of the ten Regions of the country, a pilot programme aimed at performing routine drug susceptibility testing (DST) for previously treated PTB cases. The objectives of the programme were to evaluate the feasibility of monitoring drug resistance among retreatment cases under programme conditions and to measure the presence and magnitude of anti-TB drug resistance in order to inform NTP policies.

**Findings:**

This retrospective cohort study was conducted in the Littoral Region of Cameroon in 2009. It included all sputum smear positive (SM+) PTB cases registered for retreatment. TB cases were identified and classified according to World Health Organization (WHO) recommendations for national TB programs. Bacterial susceptibility testing to first-line anti-TB drugs was performed using standard culture methods. In 2009, 5,668 TB cases were reported in the Littoral Region, of which 438 (7.7%) were SM + PTB retreatment cases. DST results were available for 216 (49.4%) patients. Twenty six patients (12%) harbored multi-drug resistant (MDR) strains. Positive treatment outcome rates were particularly low in retreatment patients with MDR-TB (46.2%; 95% CI: 27.1-66.3). Thirteen MDR-TB patients were treated using a standardized MDR treatment regimen. Delivery of laboratory results took on average 17 (12-26) weeks.

**Conclusions:**

WHO-recommended routine DST in retreatment patients seems feasible in Cameroon. However, coverage needs to be improved through better management. Moreover, diagnostic delay should be shortened by introducing more rapid diagnostic tools. The high risk of MDR in standard regimen failure cases virtually rules out the standard retreatment regimen for such patients without prior DST.

## Findings

In 2009, about 30,000 or 11% of estimated overall 500, 000 MDR-TB cases were enrolled for second-line treatment. Achieving universal access to diagnosis and treatment of multidrug-resistant and extensively drug-resistant tuberculosis (M/XDR-TB) remains a challenge [1,2]. According to WHO, the surveillance and early diagnosis of drug-resistance in TB is ideally carried out by routine drug-susceptibility testing (DST) of all TB patients [3]. Where the circumstances or means do not permit systematic assessment of all cases with a new episode of TB, alternatively, at least patients known to be at higher risk of carrying drug-resistant strains such as previously treated patients should be assessed systematically. The Global Plan to STOP TB (2006-2015) foresees that by 2015 all countries should carry out DST for all retreatment TB patients [4].

In Cameroon, in 2009, the estimated TB incidence rate was 182 cases per 100,000 inhabitants [1]. TB patients are treated under the National TB Control Programme (NTP) according to the NTP guidelines and WHO-recommended standard regimens [5]. The total number of incident MDR-TB cases is estimated to be 440 per year. The cumulative number of MDR-TB patients put on treatment during the period from January 2005 to December 2008 in either of the two national MDR-TB treatment centres was 109 [6]. Since 2008, patients are treated free-of-charge following a standardized 12-month treatment scheme comprising gatifloxacin (Gfx), clofazimine (Cfz), ethambutol (E), prothionamide (Pto) and pyrazinamide (Z), administered throughout the 12 months of treatment and supplemented during a minimum four months intensive phase by kanamycin (Km) and isoniazid (H) (4KmGfxPtoHCfzEZ/8GfxPtoCfzEZ). Preliminary results of this standardized short-course regimen appear to be very promising [7]. Due to the continuous reporting of MDR-TB among previously treated patients and, in particular, among standard regimen failure cases, and following the establishment of a supervised Regional TB Reference Laboratory with culture capacity, a pilot programme aimed at performing routine DST among previously treated smear microscopy positive pulmonary TB (SM + PTB) cases was implemented under programme conditions in the Littoral Region, one of the 10 Regions of Cameroon. The objectives were to evaluate the feasibility of monitoring drug resistance among retreatment cases under programme conditions, measure the presence and magnitude of drug resistant and particularly MDR-TB in an important subset of retreatment TB patients in Cameroon and discuss possible implications of the results for NTP policies.

## Materials and methods

### Setting and study population

The Littoral Region has about 3.5 million inhabitants, with some 3 million people living in the economic capital, Douala. TB cases are reported and virtually treated exclusively within a network of 29 functional government or private confessional Basic Management Units (BMU). The number of TB patients treated in the private sector is negligible as anti-TB drugs are not available, neither in private pharmacies nor through drug vendors in the informal sector. Twenty of the BMUs are located in Douala and receive about 75% of the Littoral Region's annual incident cases. More than ninety-five per cent of reported cases originate from the Littoral Region. One of the two MDR-TB treatment centres is situated in Douala. Provider initiated voluntary counselling and testing for HIV with consequent Co-trimoxazole prophylaxis is offered free-of-charge to all newly diagnosed TB-cases. HIV + patients are systematically referred to the nearest HIV/AIDS treatment service for further evaluation of their infection.

### Inclusion criteria

This study included all consecutively registered SM + PTB retreatment cases with a new episode of TB admitted any one the 29 BMUs of the Littoral Region in Cameroon between 1 January and 31 December 2009 for a standardized retreatment regimen according to NTP guidelines, categorized as "Relapse", "Treatment failure" or "Return after default" (WHO case definitions), and for whom DST results were available [3].

### Exclusion criteria

The study excluded all "Other retreatment" cases, i.e. cases with unknown previous treatment outcome, patients returning to treatment with smear-negative PTB or bacteriologically not confirmed extrapulmonary TB as well as cases with non-tuberculosis mycobacteria.

### Transportation of specimen and bacteriological procedures

TB laboratory technicians in all BMUs had been instructed to keep one fresh morning sputum specimen of all consecutively notified SM + PTB retreatment refrigerated and to ensure their transportation within 72 hours to the Regional TB Reference Laboratory in Douala. Here, a new smear was made after decontamination of the specimens and concentration by centrifugation. According to routine procedures, two slopes of Löwenstein-Jensen (LJ) medium per specimen were inoculated and incubated at 37°C, discarding negative slopes after ten weeks. Positive cultures were then transported to the national TB Reference Laboratory, Centre Pasteur du Cameroun (CPC), for DST using the indirect proportion method on LJ medium [8]. The following drugs were tested: rifampicin (R), H, E, streptomycin (S), Km, and Gfx. - External quality assessment of the Regional TB Reference Laboratory in Douala is assured through quarterly supervisions by CPC personnel, and through continuous feedback on quality of cultures sent to CPC for DST. The CPC is part of the Supranational Reference Laboratory Network of the Institute of Tropical Medicine in Antwerp and is subjected to bi-annual external quality assessments. - Patients and BMU were informed about DST test results. A standardized MDR-TB treatment was proposed to all patients identified with MDR-TB without clinical improvement under retreatment and still sputum smear positive at their five-month control.

### Ethics

The study protocol was reviewed and approved by the competent administrative authorities and the National Ethics Committee of Cameroon.

### Statistical analysis

TB and demographic data, as well as HIV status, were extracted from the NTP standard TB registers. TB culture and DST results (including delivery dates) were tracked, respectively, through the Regional and National TB Reference laboratories. Culture and DST results were matched with patients registered in the BMUs using the date of registration and the patient's registration number. Main outcome measures were the proportion of reported retreatment cases with DST results available, the proportion of MDR-TB per subcategory of retreatment cases, and the proportion of retreatment cases identified with MDR-TB and treatment success defined as "Cured" or "Treatment completed". Double data entry with data cleaning and analysis was done in Epi Info 6.04 d. Per category data was expressed in percentages, continuous variables as mean and standard variation. All proportions were compared using the *χ*^2 ^test statistic and Fisher exact test where appropriate.

## Results

**Figure 1:**
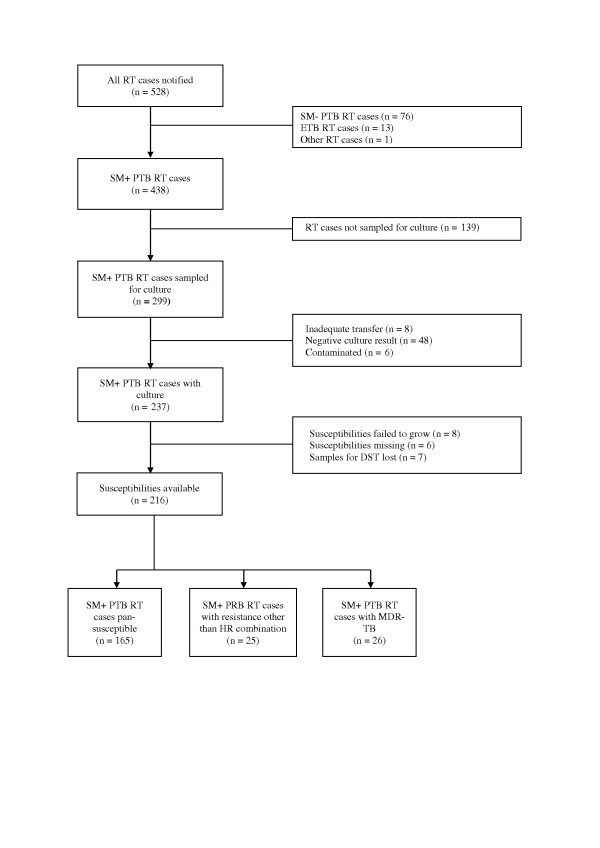
**Flow chart presenting the study population**. (SM+/SM- PTB = smear positive/smear negative pulmonary tuberculosis; DST = drug susceptibility testing; ETB = extra pulmonary tuberculosis; MDR-TB = multidrug resistant tuberculosis; RT = retreatment).

In 2009, 5,668 TB-cases were reported in the Littoral Region, of which 528 retreatment cases (9.3%). Of this number, 438 (83.0%) were SM + PTB retreatment cases registered in any of the three sub-categories (relapse, treatment failure, return after default). Where cultures were negative, failed to grow, were contaminated or where data were missing, patients were excluded as TB could not be confirmed. DST results were available for 216 (49.4%) SM + PTB retreatment cases. The selection of the study population is shown in Figure 1. - The majority of patients were males (71.3%). Female patients were significantly younger than male patients (29.9, [SD +/- 7.5] vs. 38.5 [SD +/- 12.2], respectively; *p *< 0.001). HIV infection rate was 25.9% and slightly higher among female patients. Table 1 presents the sampling coverage with positive culture and DST results per retreatment subcategory. The proportions for sampling coverage, positive culture results and DST results did not differ significantly between the three subcategories. Equally, the population with DST results did not differ proportionally from the source population (total of SM + PTB retreatment patients of the region) in terms of sex and age distribution.

**Table 1:** DST coverage of reported SM + PTB RT-cases by subcategory in the Littoral Region, 2009

SM + PTB RT-cases, by subcategory	Relapse (n = 286) n (%)	Treatment failure (n = 23) n (%)	Return after default (n = 129) n (%)	Total (n = 438) n (%)
Sampled for culture	204 (71.3)	19 (82.6)	83 (64.3)	299 (68.3)

Positive culture	157 (54.9)	11 (47.8)	69 (53.5)	237 (54.1)

DST result	140 (49.0)	10 (43.5)	66 (51.1)	216 (49.3)

Table 2 presents the susceptibility patterns related to retreatment subcategory. A total of 26 (12%) retreatment cases with DST harbored MDR strains, with an important proportion among failure cases (70%; 95% CI: 38.0-91.7) and a significant proportion among relapse cases (12%; 95% CI: 7.5-18.4). The proportion of female patients with MDR-TB was twice that of male patients (14/62 and 12/154, respectively); OR: 2.35 (1.01-5.48). Four (15.4%; 95%CI: 5.1-31.1) among the 26 MDR-TB retreatment cases were HIV positive, of which three females. Positive sputum control exam results at month 3 were positively associated with MDR-TB (OR: 6.03, 95% CI: 1.05-32.04). HIV infection, however, was not significantly associated with MDR-TB disease.

**Table 2:** Susceptibility patterns according to retreatment subcategory in the Littoral Region, 2009

	Relapse (n = 140) n (%)	Treatment failure (n = 10) n (%)	Return after default (n = 66) n (%)	Total (n = 216) n (%)
Any resistance	34 (24.3)	8 (80.0)	9 (13.6)	51 (23.6)*

Monoresistance	14 (10.0)	0	6 (9.0)	20 (9.3)

Polyresistance other than MDR	1 (0.7)	1 (10.0)	3 (4.5)	5 (2.3)

MDR-TB	17 (12.1)	7 (70.0)	2 (3.0)	26 (12.0)

• *P *= < 0.001

Positive treatment outcome rates did not differ significantly between pansusceptible, monoresistant, and polyresistant patients. However, only twelve out of 26 MDR-TB patients (46.2%; 95% CI: 27.1-66.3) had a successful treatment outcome due to a high failure rate (42.3%; 95% CI: 24.0-62.8) (Table 3). No statistically significant association was observed between HIV co-infection and unfavorable treatment outcomes (death, failure, unknown outcome).

**Table 3:** Treatment outcomes of SM + PTB retreatment cases by subcategory according to drug susceptibility patterns in the Littoral Region, 2009

	Pansusceptible (n = 165) n (%)	Monoresistant (n = 20) n (%)	Polyresistant (n = 5) n (%)	MDR (n = 26) n (%)	Total (n = 216)
Treatment success*	115 (69.7)	16 (80.0)	3 (66.6)	12 (46.2)	146 (67.6)

Treatment failure	2 (1.2)	0	1 (16.7)	11 (42.3)	14 (6.4)

Died	5 (3.0)	0	0	0	5 (2.3)

Defaulted	13 (7.9)	2 (10.0)	0	3 (11.5)	18 (8.3)

Transferred	30 (18.2)	2 (10.0)	1 (16.7)	0	33 (15.3)

*) Treatment success = cured or treatment completed

Delivery of laboratory results to clinicians took on average 17 (12-26) weeks. Of the 26 diagnosed MDR-TB cases, 13 patients were treated following the standardized MDR treatment regimen; three patients died before the start of MDR-treatment; four patients did not accept another treatment; four patients remained smear-negative up to one-year follow-up; two patients were lost to follow-up, one during retreatment, the other after having being declared cured.

## Discussion

In our retrospective cohort study, 51% of SM + PTB retreatment patients showed resistance to one or more anti-TB drugs; those having failed the standard regimen showed at base-line a high rate of MDR-TB; this rate was also important in relapse cases. It cannot be excluded that the relative high failure rate for the retreatment regimen is partly due to primary MDR-TB with subsequent transmission. Recently published data from other Regions of Cameroon suggest an increase in the rate of primary MDR-TB [9]. As predictable, the proportion of MDR-TB patients with unfavorable treatment outcome after standardized chemotherapy, in line with the WHO-recommended retreatment regimen, was unacceptably high. Finally, the delay between sampling and availability of laboratory results, 17 weeks on average, was too high for timely treatment decisions.

The study has several limitations, the most important of which is possible selection bias with about half of the cohort excluded due to missing DST results. The incomplete coverage of the target population may have distorted the findings. Yet, all BMUs participated in sampling and the proportion of retreatment subcategories, age and sex distribution, as well as the HIV co-infection rate in the sampled part of the study population and the full target population were comparable. - Secondly, our study was records-based and patients' classification was not controlled. In particular, misclassification of relapse cases as new cases is common under programme conditions; it is possible that the proportion of relapse cases was underestimated. On the other hand, the proportion of retreatment cases with their different subcategories, as well as drug resistance profiles, confirm trends observed in the West Region (2004/05, unpublished data), the Littoral Region (2008), and the North-West Region (2009) [10,11]. - Finally, the exclusion of DST testing results of smear-negative PTB retreatment cases in our analysis might have to a certain extent changed the overall anti-TB drug resistance profile in our cohort.

As previously described for another Region in the country, we did not observe any association between MDR-TB and HIV [12]. This corroborates the findings of a recent review which did not find any evidence supporting a systematic association between MDR-TB and HIV infection across time and geographic locations in Sub-Saharan African countries [13].

Due to very long turn-around times for DST results (> 4 months) with two different laboratories involved and frequent subculturing needed before obtaining DST results, the time for initiating MDR-TB treatment in practice coincided with the 5-month control period. For patients with laboratory-proven MDR-TB but having responded favorably to the standardized retreatment regimen, no MDR treatment was initiated, thus trading off the benefits of a MDR-TB treatment against the fact that the standard retreatment scheme may give positive treatment outcomes and against the possible side effects of the MDR-TB treatment, in particular a prolonged treatment with potentially ototoxic aminoglycosides. Indeed, in our cohort, four (15%; 95% CI: 5-38) MDR-TB patients who were cured presented no recurrent disease (microscopy and culture negativity) for up to one year after the end of their retreatment. This clinical (and ethical) decision is debatable [14].

For almost two decades, country and multi-country studies have gathered overwhelming evidence on unfavorable outcomes in patients failing the standard regimen, mostly those infected by MDR-TB and treated according to the standard WHO-recommended retreatment regimen [15-20]. Two recent follow-up studies evaluating this regimen observed, even after cure, very high rates of recurrence in TB patients with MDR-TB at base-line [21,22]. Notwithstanding, most TB programmes in low-income countries in sub-Saharan Africa treat, empirically, every year 10-20% of their TB patients using this regimen [2,15,19]. The design of NTP retreatment regimens depends on the epidemiological context, the program's performance, and the means available. However, MDR-TB treatment should no longer be a heavily conditioned option; it is a must, for public health, as well as human rights reasons [23]. Extrapolating prudently the results of our study to the rest of the country, a recommendation for the diagnosis and treatment strategy for retreatment patients could presently be formulated as follows: 1. Rapid scaling-up of a well-managed collection and transportation system in view of systematic DST for at least all failure and relapse cases [24]; 2. Initiation of standard retreatment regimen for defaulters and relapse cases, pending DST results; if results indicate, change to standardized MDR-TB regimen; 3. Systematic DST for standard regimen failure cases before initiating treatment, then standardized retreatment regimen or MDR-TB treatment; in order to reduce the 'diagnosis window period', the NTP should set up, without delay, newer and more rapid tuberculosis diagnosis technologies (either line probe assays or Xpert^® ^MTB/RIF) at selected strategic sites to enable early detection of MDR resistance and prioritize high risk patient groups such as TB patients having failed the standard regimen [25].

## Conclusion

Preliminary results of systematic DST for SM + PTB retreatment patients showed that early detection of MDR-TB under program conditions in Cameroon might be feasible if more rapid diagnosis tools required for initiating adequate treatment timely are put in place. Data also suggests that a standardized retreatment regimen is inadequate in Cameroon for failure cases and that there is a significant proportion of relapse cases. Systematic DST for retreatment patients should be implemented country-wide, while improving access to rapid diagnosis.

## Abbreviations

BMU: Basic Management Unit; CI: Confidence interval; CPC: Centre Pasteur de Cameroun; DST: Drug susceptibility testing; E: Ethambutol; Gfx: Gatifloxacin; H: Isoniazid; HIV: Human immunodeficiency virus; Km: Kanamycin; MDR: Multi-drug resistant; NTP: National tuberculosis control program; OR: Odds ratio; PTB: Pulmonary tuberculosis; Pto: Prothionamide; R: Rifampicin; S: Streptomycin; SD: Standard deviation; SM+: Sputum smear positive; TB: Tuberculosis; XDR: Extensively drug resistant; WHO: World health organization; Z: Pyrazinamide.

## Competing interests

The authors declare that they have no competing interests.

## Authors' contributions

JN and NV drafted the manuscript; EF and JLA monitored the data collection, participated in the data analysis, and contributed to the drafting of the manuscript. All authors read and approved the final manuscript.
